# Systematic Review of Immunosuppression After Chimeric Antigen Receptor T-Cell Therapy for Posttransplant Lymphoproliferative Disorder

**DOI:** 10.1016/j.ekir.2026.106367

**Published:** 2026-02-16

**Authors:** David Synnott, Adam Bowden, Donal J. Sexton

**Affiliations:** 1 Renal Unit, St James Hospital, Dublin, Ireland; 2 Trinity Kidney Centre, School of Medicine, Trinity College Dublin, Dublin, Ireland; 3 Trinity St James’s Cancer Institute, St James’s Hospital, Dublin, Ireland

**Keywords:** CAR T-cell therapy, lymphoma, onconephrology, PTLD, transplant

## Abstract

**Introduction:**

The use of chimeric antigen receptor (CAR) T-cell therapy for relapsed or refractory posttransplant lymphoproliferative disorder (PTLD) in solid organ transplant recipients is a rapidly evolving frontier in immunotherapy and transplantation. In this context, clinicians must carefully balance the competing risks of allograft rejection, the potential for maintenance immunosuppression to diminish CAR T-cell efficacy and treatment response, alongside an increased infection risk. Given the heterogeneous nature of PTLD and the scarcity of clinical trial data in this specific population, there is currently no established consensus regarding the optimal maintenance immunosuppressive strategy post–CAR T-cell therapy.

**Methods:**

To address this gap, we conducted a systematic review of published data pertaining to CAR T-cell therapy and PTLD after solid organ transplantation.

**Results:**

Our findings reveal substantial variability in immunosuppressive management before and after CAR T-cell infusion, including the withholding of immunosuppression, or the use of single agent or combinations of corticosteroids, calcineurin inhibitors (CNIs), antimetabolites, or mammalian target of rapamycin (mTOR) inhibitors. Given the limited data to date, balancing these competing risks is challenging, and no single immunosuppression regimen has emerged as clearly superior.

**Conclusion:**

Our review underscores the necessity of an individualized approach to these patients that accounts for factors such as CAR T-cell persistence and prolonged B-cell depletion, and highlights the need for further research on this clinical scenario.

## Graphical abstract

**Figure:**
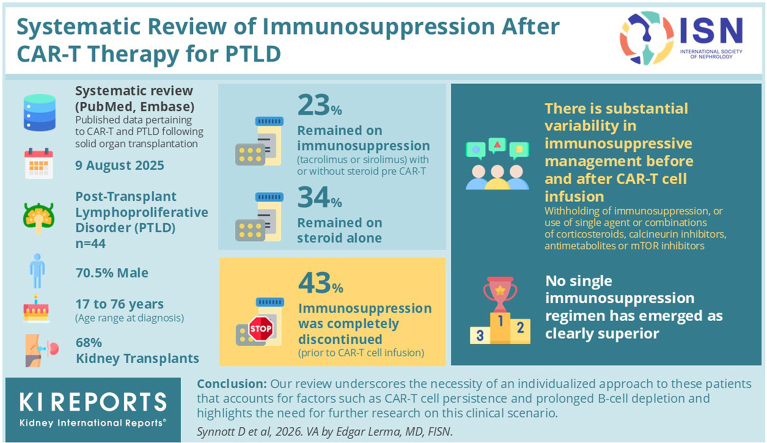

PTLD was first identified in 1984, encompassing a spectrum of lymphoproliferative diseases that arise as a complication of bone marrow or solid organ transplantation.^1^ Lymphomas can account for 20% of posttransplant cancers in patients undergoing solid organ transplantation, making PTLD one of the most prevalent malignancies in this population.^2^^,^^3^ Impaired immune surveillance because of immunosuppressive therapy, coupled with the activation of oncogenic viruses, particularly Epstein-Barr virus (EBV), plays a critical role in the development of PTLD.^4^ Overall, the mortality rates of PTLD have been estimated to be between 30% and 60%,^5^ underscoring the critical need for improved treatment strategies, which remain an active area of ongoing research. CAR T-cell therapy targeting CD19 has shown significant efficacy across various subtypes of relapsed or refractory non-Hodgkin lymphoma; an increasing number of patients with multiple comorbidities, including a small number of solid organ transplant recipients, are now receiving this therapy worldwide.^6^ In relapsed or refractory aggressive B-cell non-Hodgkin lymphomas, all currently approved CAR T-cell products are autologous, CD19-directed therapies. These include axicabtagene ciloleucel, tisagenlecleucel, lisocabtagene maraleucel, and brexucabtagene autoleucel, which have demonstrated high response rates and durable remissions in pivotal phase 2 trials in large B-cell lymphoma and mantle cell lymphoma.^7^, ^8^, ^9^, ^10^ CD19 is a pan–B-cell marker expressed on normal and malignant B cells and is typically retained in B-cell PTLD, even when CD20 or surface Ig are downregulated, making CD19 a biologically attractive target in this population.^11^

Although some promise is emerging in the *in vivo* creation of CAR T-cell therapy, at present, the process involves drawing blood from the patient by leukapheresis to isolate native T cells. These T cells are then genetically modified using a disarmed viral vector to express CARs on their surface. The engineered CARs enable T cells to specifically recognise and bind to a target protein, such as tumor-associated antigens, and these modified T cells are subsequently expanded into hundreds of millions within a specialized cell manufacturing facility. After the patient receives a lymphodepleting chemotherapy regimen (commonly referred to as the conditioning regimen), the CAR T cells are infused into the patient, where they selectively target and eliminate cells expressing the specific tumor antigen.^12^^,^^13^ Recent reviews demonstrate that fludarabine-cyclophosphamide is the dominant regimen used before CD19 CAR T-cell therapy and functions as the conditioning backbone.^14^^,^^15^ In all of the cases identified in this review, where a lymphodepleting regimen is specified, fludarabine and cyclophosphamide are consistently the agents used.

The use of CAR T-cell therapy and reduction or withdrawal of maintenance immunosuppression in the solid organ transplant cohort, however, carries a risk of allograft rejection.^16^ Furthermore, transplant immunosuppressive therapy may reduce the effectiveness of CAR T cells because of their direct impact on T-cell development, activation, proliferation, and persistence.^17^ Establishing the ideal immunosuppressive regimen for patients undergoing CAR T-cell therapy for PTLD therefore presents a unique challenge in transplantation. There is currently no consensus or prospective data on how best to balance the risks of allograft rejection and potentially compromising CAR T-cell efficacy when using exogenous maintenance immunosuppression. Consequently, reported cases have been managed on an individualized basis within multidisciplinary transplant–hematology teams, and prospective systematically collected data are needed to inform future standardized approaches. The heterogeneous nature of PTLD as well as the lack of clinical trials in this area and in the increasing use of CAR T-cel therapy in PTLD represents an emerging clinical dilemma. To enhance the understanding of the potential immunosuppressant strategies after CAR T-cell therapy, we conducted a systematic review of the existing literature to identify the totality of published clinical experience to date and describe the literature pertaining to the postulated relative suppressive impacts on CAR T cells *in vivo*. Accordingly, we present the conclusions of this review as opinions and considerations to inform the design of future prospective studies, rather than definitive practice standards.

## Methods

### Search Strategy

**Figure 1:**
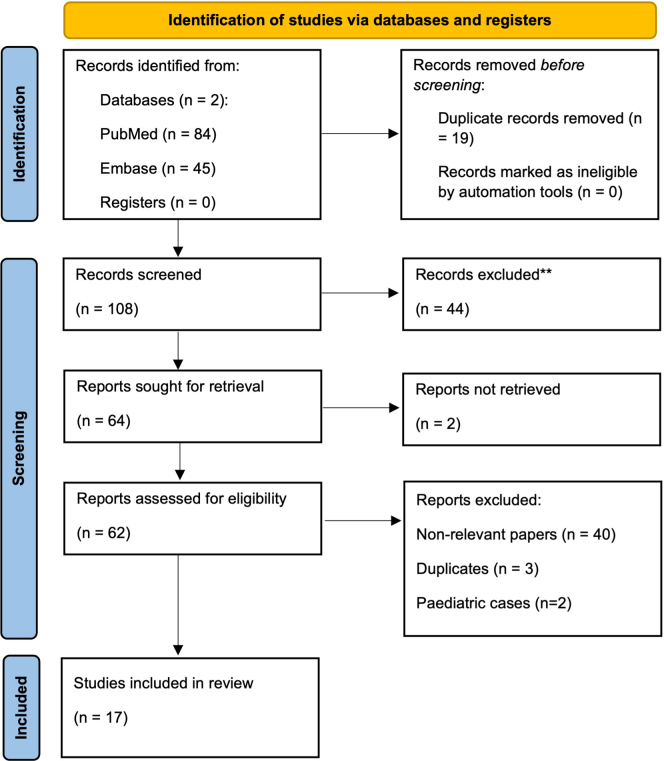
PRISMA 2020 flow diagram for systematic review of chimeric antigen receptor T-cell therapy for post-transplant lymphoproliferative disease in solid organ transplant recipients.

We approached this search using systematic review methodology, looking for the literature pertaining to CAR T-cell therapy in patients with PTLD and aiming to identify immunosuppressive strategies for transplant patients’ post CAR T-cell therapy. Searches were performed on PubMed and Embase databases on August 9, 2025. The search strategy involved using various search terms for the concepts of “solid organ transplant,” “PTLD,” and “CAR T cell therapy” and then combining the search for all 3 concepts using Boolean operators. We did not limit the publication date or article type in our search; however, results generated were filtered to articles in English only. Following the detailed search, 2 reviewers (DS and AB) independently screened article titles and abstracts before subsequently conducting a screen of full texts of those articles deemed to be possibly relevant. Case reports, case series, retrospective cohort studies, meta-analyses, and other systematic reviews were included. Non–peer-reviewed articles and pediatric cases (subjects aged < 16 years) were excluded. Duplicated case reports (i.e., case reports describing identical cases with matching institutional affiliations) were identified and removed. The search yielded 17 articles suitable for inclusion in this literature review (Figure 1). Within these identified articles, a further paper of interest was identified through backward and forward citation tracking. Data pertaining to patient and disease characteristics, lines of PTLD treatment, adjustments to immunosuppressive regimens, CAR T-cell therapy complications, disease, and graft outcomes were extracted.

## Results

In Table 1,^18^, ^19^, ^20^, ^21^, ^22^, ^23^, ^24^, ^25^, ^26^, ^27^, ^28^, ^29^, ^30^, ^31^, ^32^, ^33^ we summarize the current literature describing cases of CAR T-cell therapy for PTLD.

**Table 1:** Current literature describing cases of CAR T-cell therapy for solid organ posttransplant lymphoproliferative disorder

Author/ Publication type/ Number of subjects	Solid organ transplant	Age, yr/Sex	Lymphoma histology + subtype	EBV status IPI and Stage	Immunosuppression at diagnosis	Adjustment to immunosuppression (pre CAR-T)	Lines of therapy	Bridging therapy	CAR T cell product	Complications (including ICANS/CRS)	Post CAR-T Immunosuppression	Graft outcome	Disease outcome post CAR-T
Dang *et al.*^18^ Case report 1	Heart	17/F	mPTLD (DLBCL Non-GCB)	EBV ve+ IPI NS Stage III	Tacro, MMF, prednisolone	MMF stopped. Tacro reduced (trough 1–2 ng/ml) Prednisolone (5 mg) maintained	R-COP ×2, R-COPADM ×2, R-CYVE ×2, O-ICE ×2 + radiation, EBV directed CTLs	RT	Axi-cel	Grade 1 CRS Severe headaches/vasoconstrictive syndrome MAC pulmonary infection Pancytopenia	Tacro and prednisolone continued	No graft dysfunction	CR at 6 mos
De Nattes *et al.*^16^ Case report 1	Kidney	40/F	Burkitt-like	EBV ve+ IPI NS Stage NS	Tacro and AZA	AZA stopped. Tacro stopped post–first line treatment. Prednisolone (20 mg) stopped preleukapheresis	Rituximab ×4, R-COPADM, R-CYM, R-ICE, ASCT	Rituximab and dexamethasone	Tisa-cel	Grade 1 CRS Pancytopenia	Steroid reintroduced D10, subsequently stopped D35 and then restarted with graft rejection	Acute T cell mediated rejection D81 Graft function improved with steroid	POD at 6 mos, died following salvage rituximab and lenalidomide
Feng *et al.*^19^ Case report 1	Kidney	46/M	DLBCL GCB	EBV ve-IPI 4 Stage IV	Tacro (4mg), MMF, Prednisolone (20 mg)	MMF stopped. Tacro maintained at reduced dose (1 mg) Prednisolone reduced to 10 mg and stopped day of leukapheresis	R-CHOP x2, R-ECHOP x2, R-BEVD + R-ICE	PD1 inhibitor (Sintilimab) - continued post–CAR-T	Not specified	Grade 1 CRS Grade 3 ICANS	Tacro 1 mg, decline in renal function with attempt to reduce to 0.5 mg at 4 wks	Acute rejection on attempt to reduce tacrolimus dose	PR for 18 wks, followed by POD
Hernani *et al.*^20^ Case report + Literature review 1	Kidney	56/M	DLBCL GCB	EBV ve-IPI 3 Stage IV	Tacro, MMF, Prednisolone (5 mg)	Tacro and MMF switched to Everolimus (1.25 mg/24 h), stopped pre–CAR-T infusion. Prednisolone maintained at physiological dosing	R-CHOP ×6, R-ICE ×2, R-GemOx ×4, PBR ×5, RT	Cyclophosphomide ×2 (500 mg/m^2^ D1) + Prednisolone (60 mg/m^2^ D1–5)	Axi-cel	Grade 1 CRS Grade 2 ICANS Septic shock, Pseudomonas pneumonia Pancytopenia CMV reactivation	Prednisolone discontinued at 2 mos	Proteinuria (0.6 g) at 6 mos, improving with ACE inhibitor. Creatinine stable	CR at 1 and 6 mos
Hickmann *et al.*^21^ Case report 1	Heart	68/M	mPTLD (DLBCL)	EBV ve+ IPI NS Stage NS	Tacro, MMF, prednisolone	MMF stopped at diagnosis. Tacro stopped 1 wk preleukapheresis, restarted and subsequently stopped prelymphodepletion. Prednisolone dose increased	Rituximab ×4, R-CEOP ×2	R - GemOx	Axi-cel	Grade 1 CRS Grade 1 ICANS	Tacro restarted D30	Not specified	CR at D100
Ibrahim *et al,*^22^ Case series 5	Liver	36/M	mPTLD (DLBCL Non-GCB)	EBV ve-, cMYC ve+ IPI/Stage NS	Tacro, MMF	MMF stopped. Tacro subsequently stopped with disease progression	R-ICE×2, ASCT	None	Axi-cel	Grade 1 CRS	Tacro resumed 4 mos post–CAR T	Liver rejection at 11 mos because of poor Tacro adherence, rescued with MMF/prednisolone. Continued on Tacro + prednisolone	CR at 3 yrs
	Liver	76/M	mPTLD (DLBCL GCB)	EBV NS IPI/Stage NS	Tacro	Tacro held 5 d before to leukapheresis	R-mini CHOP ×2, R-CHOP ×2, R-GemOx ×2	None	Axi-cel	Grade 2 CRS	Tacro resumed D36	Moderate acute rejection, resolved with reintroduction of Tacro	POD and death at 7 mos
	Kidney	62/M	mPTLD (HBCL)	EBV NS, IPI/Stage NS	Tacro + Prednisolone (5 mg)	Tacro stopped following leukapheresis Prednisolone increased to 10 mg	R-CHOP ×3, IVAC	Gemcitabine + Oxaloplatin ×2	Axi-cel	Grade 3 CRS Grade 2 ICANS	Remained on 10 mg prednisolone	No graft dysfunction	CR at 3 mos and 3 yrs
	Kidney	35/M	mPTLD (HBCL)	EBV NS, IPI/Stage NS	Everolimus + Prednisolone (10 mg)	Everolimus stopped before leukapheresis. Pred increased to 40 mg for ×1/52, then 20 mg ×3/7before leukapheresis. Then increased to 40 mg as a bridge to CAR-T, then reduced to 20 mg before lymphodepletion	R-EPOCH ×1, R-CHOP ×5, Lenalidomide salvage + RT, Rituximab/Bortezomib/ibrutinib/methylprednisolone	None	Axi-cel	Grade 1 CRS	Pred reduced to 10 mg at D30 because of mixed response, then to 5 mg because of POD at 3 months	Not specified	POD at 3 mos. Additional lines of therapy inc. polatuzumab, rituximab and pembrolizumab - PR at 12 mos
	Kidney	54/F	mPTLD (HBCL Non-GCB)	EBV ve-IPI/Stage NS	Everolimus + Prednisolone	Everolimus stopped. 2 wks before leukapheresis, Pred increased to 20 mg	R-CHOP ×4, Rituximab infusions, Rituximab/Carboplatin/etoposide salvage	Palliative RT	Manufacturing unsuccessful	N/A	N/A		Rapid POD
Kline *et al.*^23^ Case report 1	Kidney	55/F	mPTLD (DLBCL)	EBV NS IPI NS Stage NS	MMF, Tacro + Prednisolone	MMF stopped. Tacro switched to sirolimus - held 19 d before leukapheresis, restarted and then held before CAR-T infusion. Prednisolone tapered before leukapheresis	R-CHOP ×6 (vincristine omitted for ×3 cycles because of neuropathy), PBR	RT	Axi-cel	Nil	Prednisolone 10 mg restarted D7	Not specified	Died at 2 mos from SARS COV 2 infection
Krishnamoorthy *et al.*^24^ Case series 3	Kidney - pancreas	54/M	DLBCL	EBV ve-IPI 2 Stage IV	AZA, Tacro, Prednisolone	AZA stopped Low dose Tacro (1.5 mg) + Prednisolone (5 mg) continued	Rituximab monotherapy, R-CHOP ×6 ,R-ICE ×3	None	Axi-cel	Grade 1 CRS, Grade 2 ICANS, PUO, delayed grade 3 ICANS	All immunosuppression stopped at D30	No renal dysfunction	Refractory disease. Died D115
	Heart	54/F	DLBCL	EBV ve-IPI 3 Stage NS	MMF, Tacro, Prednisolone	Tacro switched to Sirolimus. MMF and prednisolone changes not specified	R-CHOP ×5, R-ICE ×2	Lenalidomide	Axi-cel	Grade 2 CRS, Grade 3 ICANS, AKI requiring RRT, Lower GI bleed	Not specified	Not specified	POD, died D44
	Kidney	71/M	DLBCL	EBV ve-IPI 4 Stage NS	AZA, Tacro, Prednisolone	AZA and Tacro stopped	R-CHOP ×1, R-DHAX salvage, ibrutinib	Gemcitabine, etoposide	Axi-cel	Grade 3 CRS, Grade 4 ICANS, AKI requiring RRT, VRE bacteremia, Aspergillus pneumonia	Not specified	Not specified	Refractory disease. Died D15
Luttwak *et al.*^25^ Case series 3	Kidney	69/M	DLBCL GCB	EBV ve-IPI 4 Stage IVB	Tacro Other agents not specified	Tacro continued. Other agents not specified	DA R-EPOCHx6, R-GDP	Polatuzumab vedotin +Rituximab + bendamustine	Tisa-cel	Grade 1 CRS, febrile neutropenia	Tacro continued (median level 4.2 ng/ml, range: 2–8 ng/ml). Others not specified	No graft dysfunction	CR at 1 month, disease relapse at 3 mos
	Kidney	50/F	DLBCL GCB	EBV ve-IPI 4 Stage IV	Tacro Other agents not specified	Tacro continued. Other agents not specified	R-CHOPx6, R-ICE + ASCT	Gemcitabine-based	Tisa-cel	Grade 2 CRS, pancytopenia	Tacro continued (median level 4.1ng/mL, range 1.3-16 ng/mL). Others not specified	No graft dysfunction	CR at 9 months
	Liver	66/M	DLBCL Non-GCB	EBV ve-IPI 4 Stage IV	Tacro only	Tacro continued	R-CHOPx6, ICE	RT only	Tisa-cel	Grade 1 CRS, AKI	Tacro continued (median level 3.3 ng/ml, range: 2–7 ng/ml)	No graft dysfunction	PR at 1 and 3 months
Mamlouk *et al.*^26^ Case series 3	Kidney	38/M	DLBCL GCB	EBV ve-IPI 3 Stage IV	MMF, Tacro, Prednisolone	MMF stopped at diagnosis. Tacro stopped 2 wks before leukapheresis. Continued prednisolone (5 mg)	R-EPOCH, R-GemOx	Not specified	Axi-cel	Grade 1 CRS, no ICANS	Prednisolone continued	Borderline cell-mediated rejection at 16 wks	Disease remission at D30
	Kidney	44/M	DLBCL GCB	EBV ve-IPI 3 Stage IV	Sirolimus + Prednisolone	Sirolimus stopped 4 wks before leukapheresis Prednisolone 20 mg reduced to 4.5 mg/d for 4 wks and then discontinued 1 wk after leukapheresis	R-CHOP ×2	Not specified	Axi-cel	Grade 1 CRS, Grade 3 ICANS, HAP, AKI	Prednisolone restarted at 5 mg/d at 8 wks	No graft dysfunction	CR at 12 weeks, followed by disease relapse at 34 weeks
	Kidney	41/M	DLBCL GCB	EBV ve-IPI 2 Stage IV	Sirolimus + Prednisolone	Sirolimus stopped 2.5 wks before leukapheresis, Prednisolone (5 mg) continued	R-EPOCH, R-GDP, R-ESCHAP, Pola-BR	Not specified	Axi-cel	No CRS or ICANS	Prednisolone continued	No graft dysfunction	POD at 12 wks
McKenna *et al.*^27^ Multicenter retrospective analysis 22 subjects	14 kidney (64%) 3 liver (14%) 2 heart (9%) 1 intestine (5%) 1 lung (5%) 1 pancreas after kidney (5%)	16 (73%) M Median age 55 (range: 28–74)	mPTLD (×20 LBCL NOS, ×1 HBCL and ×1 MCL)	18 (82%) EBV ve-1 (5%) EBV ve+ 3 (14%) unavailable IPI: 1 1 (5%) 2 5 (23%) 3 11 (50%) 4 2 (9%) 5 1 (5%) Unavailable 2 (9%) Stage I & II 2 (9%) Stage III & IV 20 (91%)	All patients on IS at time of diagnosis 20/22 > 50% reduction in dosing of IS at time of diagnosis	Immunosuppression stopped completely in 14 cases (64%) 6 (27%) continued on steroids alone 1 (5%) continued tacro + steroids 1 (5%) continued tacro alone	4 Rituximab alone 4 DA-R-EPOCH 13 R-CHOP 1 R-BENDA	1 ibrutinib + lenalidomide 1 R cytoxan/Dex 1 R Pola 1 ICE 1 Lenolidomide + Ritux 2 Gem -Ox +/- RT 2 Ritux + Etoposide/Gem 1 RT	17 (77%) Axi-cel 4 (18%) Tisa-cel 1 (5%) Brexu-cel	18 (88%) CRS: 10 (45%) grade 1 6 (27%) grade 2 1 (5%) grade 3 1 (5%) grade 4 16 (72%) ICANS: 2 (9%) grade 1 6 (27%) grade 2 6 (27%) grade 3 2 (9%) grade 4 2 (9%) TRM - sepsis + encephalopathy	Of the 8 maintained on IS: 2 Tacro + steroids 2 Tacro alone 4 steroids alone 11 of 14 remaining patients restarted IS at median 3 mos (range: 1–14 mos): 5 steroids alone 3 Tacro alone 1 sirolimus 1 everolimus	3 (14%) allograft rejections (all renal grafts, occurring at 1, 4 and 15 mos) 1 on no immunosuppression 1 on everolimus (resumed at D450) 1 on steroid (resumed D37)	12 (55%) CR 2 (9%) PR 4 (18%) stable disease 4 (18%) POD Of the 9 patients in remission at 25 mos: 2 on no IS 2 on steroids 5 on other IS (x3 tacro, x1 everolimus, x1 tacro + steroids)
Melilli *et al.*^28^ Case report 1	Kidney	40/M	mPTLD	EBV ve-IPI 2 Stage IV	Tacro, MMF, Prednisolone	Tacro switched to everolimus, subsequently stopped. MMF stopped. Prednisolone (5 mg) continued	R-CHOP ×3 R-GDP ×2 ASCT		Tisa-cel	No CRS or ICANS AKI III - query mild alloimmune rejection v AIN	Continued on prednisolone Dose increased to 1 mg/kg to treat AIN and tapered to 10 mg OD	Mild alloimmune rejection vs. acute immunoallergic interstitial nephritis	Died with POD at 3 yrs
Oren *et al.*^29^ Case report 1	Heart and Kidney	23/F	mPTLD	EBV ve+ IPI/Stage NS	Tacro, MMF, Prednisolone	MMF stopped Continued Tacro (4-6 ng/ml) Continued prednisolone (5 mg)	R EPOCH ×4 Gemcitabine + carboplatin ×2	Polatuzumab vedotin	Liso-cel	No CRS or ICANS Mild SARS COV 2 infection	Tacro/Prednisolone continued	No graft dysfunction	CR at 6 mos
Portuguese *et al.*^30^ Case report and systematic review 1 case	Kidney	47/M	mPTLD (DLBCL GCB)	EBV ve-IPI 2 Stage NS	Tacro, MMF and Prednisolone	MMF stopped Tacro stopped 41 d before leukapheresis Prednisolone (5 mg) continued	R CHOP ×6 R -ICE ×3	Polatuzumab vedotin/bendamustine/rituximab x1	Liso-cel	Grade 1 CRS AKI	Tacro restarted at D65	No graft dysfunction	Relapse at 8 mos, treated with RIS and RT - CR at 11 mos
Total 10 studies/17 patients	12 Kidney (70.6%) 2 Liver (11.8%) Heart 2 (11.8%) Pancreas after kidney 1 (5.9%)	12/M (70.6%) Median age 46 (38.5–54)	15 (88.2%) DLBCL 2 (11.8%) Burkitt lymphoma	14 EBV ve-3 EBV ve+ IPI: 2 4 (23.5%) 3 5 (29.4%) 4 5 (29.4%) NS 3 (17.6%) Stage IV 13 (76.5%) Stage 3 0 (0%) Stage NS 4 (23.5%)	Not specified	Not specified	Not specified	Not specified	11 (64.7%) Axi-cel 3 (17.6%) Tisa-cel 1 (5.9%) Liso-cel 2 others	15 (88.2%) CRS: 11 (64.7%) grade 1 3 (17.6%) grade 2 1 (5.9%) grade 3 7 (41.2%) ICANS: 1 (5.9%) grade 1 1 (5.9%) grade 2 4 (23.5%) grade 3 1 (5.9%) grade 4	Not specified	4 (23.5%) allograft rejection (all renal grafts)	10 (58.8%) CR 4 (23.5%) PR 1 (5.9%) SD 2 (11.5%) POD
Rosler *et al.*^31^ Case report + Literature review 1	Kidney	18/F	mPTLD (DLBCL Non-GCB)	EBV ve+ IPI 3 Stage IV	Tacro, MMF and prednisolone	MMF stopped Tacro and prednisolone stopped 17 d before leukapheresis	R CHOP x5 MATRix protocol	Pembrolizumab	Tisa-cel	Grade 1 CRS Grade 1 ICANS Pancytopenia	No immunosuppression	No graft dysfunction at 26 months	Ongoing remission
Veit *et al.*^32^ Case report 1	Lung	73/F	mPTLD (DLBCL GCB)	EBV ve-IPI NS Stage IV	Tacro, AZA and prednisolone	AZA stopped Tacro stopped before CAR-T infusion Prednisolone switched to dexamethasone at time of infusion	R-CHOP	RT	Axi-cel	Grade 2 CRS Grade 2 ICANS	Tacro restarted at D25	No graft dysfunction at 12 months	Ongoing remission
Panopoulou *et al.*^33^ Case series 2	Heart	24/M	mPTLD (DLBCL)	EBV ve-IPI NS Stage IV	Tacro and AZA	AZA stopped Continued Tacro	R-COP, R-COPADM ×2, R-GDP ×2, R-IVE ×2, R-ICE ×2	PBR	Axi-cel	Grade 1 CRS	Tacro continued	Not specified	PR at 3 mos, treated with RT. CR at 12 mos
	Kidney	51/M	mPTLD (DLBCL)	EBV ve+ IPI NS Stage IV	Prednisolone (20mg)	Continued prednisolone	Rituximab R-CHOP ×3 Nanatinostat R-GemOx ×2 PBR ×1	PBR + RT	Axi-cel	Grade 1 CRS	Prednisolone continued (10 mg)	Not specified	POD, died D30

ACE, angiotensin-converting enzyme; AIN, acute interstitial nephritis; AKI, acute kidney injury; ASCT, allogeneic stem cell transplant; Axi-cel, axicabtagene ciloleucel; AZA, azathioprine; Brexu-cel, brexucabtagene autoleucel; Carbo, carboplatin; CAR-T, chimeric antigen receptor T-cell therapy; CMV, cytomegalovirus; CR, complete remission; CRS, cytokine release syndrome; CTLs, cytotoxic T-lymphocytes; D: days post–CAR-T infusion; DA-R-EPOCH, dose-adjusted R-EPOCH; DLBCL, diffuse large B-cell lymphoma; DOR, duration of relapse; EBV, Epstein Barr virus; F, female; GCB, germinal center B cell; Gem, gemcitabine; HAP, hospital-acquired pneumonia; HBCL, high grade B-cell lymphoma; HD, haemodialysis; ICANS: immune effector cell-associated neurotoxicity syndrome; IPI, International Prognostic Index; IS, immunosuppression; IVAC, ifosfamide, etoposide, and cytarabine; LBCL NOS, large B-cell lymphoma not otherwise specified; Liso-cel, lisocabtagene maraleucel; M, male; MAC, mycobacterium avium complex; MCL, mantle cell lymphoma; MMF, mycophenolate mofetil; mPTLD, monomorphic posttransplant lymphoproliferative disease; N/A, not applicable; non-GCB: non–germinal center B cell; NS, not specified; O-ICE, obinutuzumab, ifosfamide, carboplatin, and etoposide; PBR, polatuzumab, bendamustine and rituximab; PET, positron electron tomography; POD, progression of disease; Pola-BR: polatuzumab vedotin, bendamustine, and rituximab; PR, partial remission; PUO, pyrexia of unknown origin; R-BENDA, rituximab and bendamustine; R-BEVD, rituximab, bendamustine, etoposide, vincristine and methylprednisolone; R-CEOP, rituximab, cyclophosphamide, etoposide, vincristine and prednisolone; R-CHOP: rituximab, cyclophosphamide, doxorubicin, vincristine and prednisolone; R-COP, rituximab, cyclophosphamide, vincristine, and prednisolone; R-COPADM, rituximab, cyclophosphamide, vincristine, prednisolone, doxorubicin and high-dose methotrexate; R-CYM, rituximab, methotrexate and cytarabine; R-CYVE, rituximab, cytarabine and etoposide; R-DHAX, rituximab, dexamethasone, cytarabine, and oxaliplatin; R-ECHOP, rituximab, etoposide, cyclophosphamide, doxorubicin, vincristine and prednisolone; R-EPOCH, rituximab, etoposide, prednisolone, vincristine, cyclophosphamide and doxorubicin; R-ESCHAP, rituximab, etoposide, methylprednisolone, cytarabine, and cisplatin; R-GDP, rituximab, gemcitabine, dexamethasone and cisplatin; R-GemOx, rituximab, gemcitabine and oxaliplatin; R-ICE, rituximab, ifosfamide, carboplatin and etoposide; RIS, reduction in immunosuppression; R-IVE, rituximab, ifosfamide, vincristine; RRT, renal replacement therapy; RT, radiation therapy; RWA, real world analysis; SD, stable disease; Tacro, tacrolimus; Tisa-cel, tisagenlecleucel; TRM, treatment-related mortality; VRE, vancomycin-resistant enterococci.

Of the 17 articles included in our literature review, there were 7 case reports alone, 2 case reports with literature reviews, 6 case series (with 17 relevant cases), 1 multicenter retrospective cohort study (with 22 cases, 5 of which were reported in other included articles), and 1 case report with a systematic review examining 10 studies (all of which were captured in our search). Five other literature reviews and/or systematic reviews were identified that contributed to the content of this article but did not contain sufficient data or any cases pertaining to CAR T-cell therapy in solid organ transplant. As such, these articles were not included in the results and final summary table or PRISMA flow chart.

Our search generated a total of 44 patients with PTLD, at least 84% (*n* = 37) of whom had diffuse large B-cell lymphoma. The remaining PTLD cases included 1 Burkitt’s-like lymphoma, 3 high-grade B-cell lymphomas, 1 mantle cell lymphoma, and 2 monomorphic PTLDs not specified. Of the patients, 70.5% (*n* = 31) were male and with an age range at diagnosis of 17 to 76 years. Most solid organ transplants were renal transplants alone (*n* = 30, 68%). Changes to immunosuppression pre– and post–CAR T-cell therapy varied between cases. Immunosuppression was completely discontinued in 19 cases (43%) before CAR T-cell infusion. Ten patients (23%) remained on immunosuppression (tacrolimus or sirolimus) with or without steroid pre–CAR T-cell therapy, whereas 15 (34%) remained on steroid alone.

Of the 19 cases in which immunosuppression was held, 4 reported timing in relation to leukapheresis. These included cessation of sirolimus 19 days before, with prednisolone tapering (*n* = 1); cessation of tacrolimus and prednisolone 17 days before (*n* = 1); discontinuation of prednisolone 1 month before (*n* = 1); and cessation of sirolimus 4 weeks before with prednisolone stopped 1 week after leukapheresis. Timing relative to leukapheresis was not specified in the remaining cases.

Post–CAR T-cell therapy, of those 19 patients on no immunosuppression, 14 restarted steroids (*n* = 8 steroids alone) and/or CNIs or mTOR inhibitors (*n* = 6). For 1 case where immunosuppression had ceased before CAR T-cell therapy, posttreatment immunosuppression was not specified.

Of those patients on immunosuppression, tacrolimus was reintroduced for 4 patients already on steroids alone. Two other patients on immunosuppression during CAR T-cell therapy, had their immunosuppression completely discontinued posttreatment. Where specified, there were 9 documented allograft rejections (20.5%) (kidney, *n* = 7; liver, *n* = 2). Of these, 3 were on no immunosuppression, 2 cases were on steroids alone, 1 case was nonadherent to tacrolimus, and 1 case occurred on an attempt to reduce tacrolimus dosing. Eight of the 9 cases of rejection occurred within approximately 3 to 16 weeks of CAR T-cell therapy, with 3 of these cases at approximately 5 weeks. One case of rejection however occurred at 15 months following CAR T-cell therapy. Almost all cases of rejection resolved with the reintroduction or increase in immunosuppression. In 6 of the 9 rejection cases, the posttransplant interval was specified and, in all such cases, they were ≥ 6 years posttransplant (mean: 14 years, range: 6–27 years).

In terms of disease outcomes post–CAR T-cell therapy, where specified, 20 cases (45.5%) achieved complete remission, 3 cases (7.5%) achieved partial remission, and 2 cases (5%) were labelled as “ongoing remission,” with follow-up intervals ranging from 3 months to 3 years.

Our review of the literature was limited by the substantial heterogeneity among published cases, and the incomplete reporting for certain cases, particularly with respect to adjustments in immunosuppression pre– and post–CAR T-cell therapy and the presence or absence of allograft rejection. Instances of unreported data, including unspecified changes in immunosuppression or graft outcomes, are included in Table 1. As noted above, there were 5 cases in the retrospective cohort study by McKenna *et al.*^27^ that were clearly identified as duplicates of cases described in other included articles and were excluded from our final analysis. Although the remaining cases reported by McKenna *et al.* did not exactly match cases in other reports, because of incomplete or inconsistent reporting, the possibility of residual duplication cannot be definitively ruled out. This limitation applies to possible overlap between cases in other articles included in this review.

### Potential Strategies for Immunosuppression in Solid Organ Transplantation Post–CAR T-Cell Therapy

It is likely that in general, the risk of allograft rejection in this cohort is lowest in the immediate phase following CAR T-cell infusion subsequent to lymphodepletion conditioning, particularly in individuals who have had previous exposure to PTLD-directed chemotherapy before consideration for CAR T-cell therapy. Existing case reports have employed heterogeneous management strategies for immunosuppression in CAR T-cell therapy as described in the results section above.

Although the management of immunosuppression in solid organ transplants has similarities standard to PTLD without CAR T-cell therapy, there are some specific differences which merit appreciation. Fludarabine, which does not form part of the routine PTLD treatment is used before CAR T-cell infusion and often results in profound lymphodepletion and immunosuppression, which is likely to provide additional degrees of immunosuppression and reduction in allograft rejection at least in the initial period. Infection risk because of degree of immunosuppression is likely to align inversely with allograft rejection risk. Additional pathophysiological considerations include the rate of reconstitution of B cells after CAR T-cell therapy and the degree to which this varies by case and likely by degree of CAR T-cell efficacy and persistence. Ongoing CAR T-cell persistence would result in ongoing B-cell depletion and augmented allograft rejection risk. Poor response, and impaired or absence of persistence of CAR T-cell therapy is likely to result in earlier reconstitution.

#### Holding Immunosuppression

Considerations for managing and altering immunosuppression occur before the initiation of the CAR T-cell therapy process. Case reports have described holding immunosuppression 1 to 4 weeks before leukapheresis to ensure that there are sufficient cells available for manufacturing and to improve T-cell viability.^17^^,^^21^^,^^34^ This is particularly relevant for patients who are maintained on tacrolimus, a CNI, which blocks the production of interleukin-2 (IL-2) and therefore T-cell proliferation. CNIs continue to be withheld during CAR T-cell infusion to allow the infused CAR T-cells to expand and exert their cytotoxic activity more effectively. CAR T-cell proliferation reaches its peak within the first 3 weeks following infusion and evidence suggests that a greater peak in CAR T-cell expansion within the first 28 days after axi-cel infusion is associated with a stronger objective response.^26^ Although withholding CNIs raises the risk of allograft rejection during this period, the lymphodepleting chemotherapy administered before CAR T-cell infusion, particularly fludarabine and cyclophosphamide may offer prolonged temporary immunosuppression by decreasing the population of alloreactive lymphocytes.^35^ Further evidence to this effect is that observation that lymphodepletion regimes with fludarabine and cyclophosphamide have been reported to improve refractory autoimmune diseases in the absence of lymphoma before CAR T-cell infusion.^36^^,^^37^ In this cohort study by Müller *et al.*,^37^ patients who received lymphodepletion but did not go on to received CAR T-cell infusion had improvements in the systemic autoimmune disease; however, over the longer-term, the autoimmune disease recurrence was higher in those who did not receive CAR T-cell therapy, suggesting more profound and prolonged lymphodepletion with CAR T-cell therapy or extensive eradication of the autoreactive cell population.^37^

Targeting CD19 with CAR T-cell therapy can lead to B-cell aplasia and hypogammaglobulinemia, which has been shown to correlate with a reduction in the risk of allograft failure in mice.^38^^,^^39^ It is possible that this same immunosuppressive effect may result in the reduction or time-limited prevention of new donor-specific antibodies to the allograft and allow for a period of immunosuppression withdrawal following CAR T-cell therapy; however, this has not been investigated to date. Systematic reviews report allograft rejection rates of 14% to 23.5% post–CAR T-cell therapy, and thus, the relationship between CAR T-cell therapy and immunosuppression in solid organ transplants is evidently nuanced and complex.^27^^,^^30^

As B cells and plasma cells begin to recover following cytotoxic depletion, the risk of allograft rejection elevates. Each decision ought to be highly individualized, and influenced by specific patient factors, the type of transplant, the immunological details of the donor and recipient match, the duration following transplant, and the response to CAR T-cell therapy. Other factors which may influence the likelihood of allograft rejection in the medium to long-term include the degree of B-cell depletion and recovery post–CAR T-cell therapy, degree of initial lymphodepletion before CAR T-cell therapy, and degree of exposure to chemotherapy before being defined suitable for CAR T-cell therapy.^40^

To date, the data pertaining to allograft rejection post–CAR T-cell therapy is mainly in case report form and case series as outlined in our systematic review (Table 1). Cases have identified a period of 8 to 12 weeks as a suitable time to consider reintroduction of immunosuppression to give sufficient time for CAR T-cell therapy to have effect,^27^^,^^30^ with others keeping this period as broad as 4 to 12 weeks.^22^^,^^26^ This is supported by the ZUMA-1 trial in patients without a solid organ transplant, which showed that maintenance of CAR T-cell response at 3 months was associated with long-term durability of response in 80% of patients with diffuse large B-cell lymphoma.^39^^,^^41^

In relation to CAR T-cell therapy use in autoimmune disease, lymphodepletion alone appears to be associated with eventual reconstitution of clonal B cells; however, CAR T-cell therapy itself to-date appears to give longer lasting remission, suggesting more protection against autoantibody production.^36^^,^^37^ In some reported cases, the autoimmune condition treated by CAR T-cell therapy have not recurred at the time of writing, which suggests a reset in autoreactivity and cure rather than remission induction.^37^^,^^42^ Whether this generalizes to donor-specific antibodies is unclear. Secondary malignancies following CAR T-cell therapy appear to be predominantly related to the exposure to the burden of chemotherapy and radiotherapy because of consideration for CAR T.^43^ For certain primary causes of end-stage kidney disease such as glomerular disease, cumulative immunosuppressive burden, and exposure to agents such as cyclophosphamide before kidney transplantation are relevant in an analogous manner. In PTLD treatment, the maintenance immunosuppression regime should consider not only the risk of organ rejection and life-threatening infection but also the risk of undertreated PTLD because of CAR T-cell suppression.

#### Corticosteroids

Corticosteroids have long been the cornerstone of immunosuppressive therapy since the advent of solid organ transplantation. Their broad antiinflammatory and immunomodulatory properties prove invaluable in both preventing and managing allograft rejection^.^^44^ Corticosteroids exert their immunosuppressive effects by antagonizing macrophage differentiation, inhibiting class 2 major histocompatibility complex antigen expression induced by interferon-γ, and suppressing the release of key cytokines such as IL-1, IL-6, and IL-2, which are critical for T and B lymphocyte proliferation and function contributing to their ability to reduce allograft rejection.^44^ Low-dose corticosteroids have been used in case studies throughout the entire CAR T-cell therapy process to mitigate the risk of graft rejection, including during leukapheresis, infusion, and thereafter.^22^ This is supported by the *post hoc* analysis of the ZUMA-1 trial cohort whereby CAR T-cell levels and response did not appear to be impacted by corticosteroid treatment.^45^ In addition, retrospective data has shown no significant differences in the distribution of central memory or exhaustion phenotypes of CAR T-cells based on corticosteroid dose, duration, or timing.^46^

Corticosteroids are not without risks. A retrospective study of 100 CAR T-cell therapy patients without solid organ transplants showed that those with higher cumulative doses of corticosteroids had significantly shorter overall and progression-free survival.^47^ The use of steroids in these cases is typically related to the treatment of immune-related toxicities of CAR T-cell therapy such as cytokine release syndrome (CRS) and immune effector cell–associated neurotoxicity syndrome, which both involve much higher doses of steroids than typical low-dose maintenance in organ transplant recipients.^48^ For solid organ transplant recipients however, corticosteroids use post–CAR T-cell infusion must be mindful to use the lowest appropriate dose and duration.

Although the use of high-dose corticosteroids and IL-6 antagonists are justified in the setting of cytokine release syndrome or immune effector cell–associated neurotoxicity syndrome, studies have suggested that high-dose corticosteroids suppress CAR T-cell expansion.^49^ However, similar to IL-6 antagonism, high-dose steroids have yet to be demonstrated to be associated with reduced disease-free survival.^49^ Nonetheless, it is likely that corticosteroids, particularly at higher doses have a suppressive effect on CAR T-cells and the risk of suppression versus CAR T-cell therapy–related side effect severity ought to be individualized. The ASCO guidelines recommend a rapid taper of corticosteroids when possible after the treatment of CAR T-cell therapy–related toxicities.^48^ In addition, the additional immunosuppressive effects of lymphodepletion preconditioning and CAR T-cell therapy itself needs to be balanced against the risk of not only organ rejection but also life-threatening infection.

#### CNIs

The data pertaining to CNI use following CAR T-cell therapy is naturally limited. One small case series has been reported of 3 patients with EBV-negative monomorphic diffuse large B-cell lymphoma PTLD refractory to chemoimmunotherapy who received CAR T-cell therapy and continued treatment with CNIs. All 3 patients achieved complete remission. Although this may suggest that CNIs may not have had a significant effect on response to CAR T-cell therapy in these specific cases, the ability to draw more generalizable recommendations is limited. A suppressive effect of the CNI on CAR T-cell therapy efficacy cannot be out-ruled based on this small series because their mechanism of action suggests a likely suppressive effect.^25^

CNIs inhibit the enzyme, calcineurin, which is a key signalling enzyme in T-lymphocyte activation.^50^^,^^51^ Cyclosporine is a prodrug that engages cyclophilin, an intracellular protein of the immunophilin family, forming a complex that then engages and inhibits calcineurin. Tacrolimus, now used more commonly than ciclosporin in solid organ transplantation, binds to FK506-binding protein 12, another immunophilin, creating a complex that inhibits calcineurin, but with superior potency. Each of these CNIs are associated with an increased risk of developing PTLD, with tacrolimus thought to increase this risk 2 to 5 times more than cyclosporine.^52^^,^^53^ Reducing the dose of CNIs by 30% to 50% is therefore recommended upon diagnosis of PTLD to reestablish T-cell function and enhance EBV-specific cellular immunity.^54^, ^55^, ^56^

In patients receiving CAR T-cell therapy, there are additional theoretical incentives to reduce or discontinue CNIs. As previously stated, CNIs block the production of IL-2 and therefore T cell proliferation. The suppression of T-cell activation by CNIs may also decrease the cytotoxic capacity of CAR T-cells, reducing their ability to kill target malignant cells, even when they can bind to the tumor antigen.

#### Antimetabolites

Although there is a lack of robust evidence with regard to the effect of antimetabolites such as azathioprine and mycophenolate on CAR T-cell function and proliferation, it is likely based on their mechanism of action that these agents will have a significant deleterious effect on proliferation of CAR T-cells *in vivo*. Azathioprine interferes with T-cell function by reducing intracellular purine synthesis, thus reducing lymphocyte numbers, and may have direct effects on activated T cells that promote apoptosis.^51^ Mycophenolic acid inhibits inosine monophosphate dehydrogenase, which preferentially affects B and T cells to reduce proliferation and suppress cell-mediated immune responses.^51^ At our institution, we aim to withhold mycophenolate at PTLD diagnosis, in advance of chemotherapy. In the paradigm of reintroduction of maintenance immunosuppression following CAR T-cell therapy, antimetabolites are not likely to be as favorable as alternative agents such as mTOR inhibitors.

#### mTOR Inhibitors

Sirolimus (rapamycin) and other mTOR inhibitors are a promising class of immunosuppressants for use after CAR T-cell therapy in PTLD. Their unique mechanisms of action provides both immunosuppressive and antineoplastic effects through inhibition of the mTOR pathway and may be less likely than CNIs or antimetabolites to hinder CAR T-cell therapy efficacy.^57^ Retrospective data suggest that in PTLD, using sirolimus where possible with CNI discontinuation, may be a viable approach for the management of patients with PTLD, because it has been associated with stable renal function, a reduced rejection rate of transplanted kidneys, and a lower cancer recurrence rate in PTLD not treated by CAR T-cell therapy.^57^

mTOR inhibitors have shown the ability to inhibit the growth of PTLD cell lines in both *in vitro* and *in vivo* studies.^58^^,^^59^ Using sirolimus after CAR T-cell therapy could possibly reduce the risk of relapse; however limited data are available on the effect of mTOR inhibitors on CAR T cells. *In vivo* mouse models suggest that the use of rapamycin does not affect the killing activity of CAR T-cells,^60^ albeit this scenario was evaluating the use of pretreatment rapamycin for acute myeloid leukemia to improve bone marrow infiltration and does not provide data on rapamycin use after CAR T-cell therapy. Another study assessed the use of sirolimus in the setting of cytopenia post–CAR T-cell infusion, where it appeared to improve the cytopenia without affecting the function of the CAR T cells, at least in the short term.^61^ Some *in vivo* and *in vitro* work on rapamycin in humanized mice and nonhuman primates suggest possible metabolic benefits to the CAR T cells that could delay or prevent exhaustion and allow more sustained action.^62^ Among the cases identified in this systematic review, we found few cases in which sirolimus was used pre– or post–CAR T-cell infusion. Mamlouk *et al.*^26^ reported 2 cases in which sirolimus was discontinued in the weeks before T-cell harvesting. Kline *et al.*^23^ outlined a case in which tacrolimus was switched to sirolimus, which was then subsequently held before cell harvesting. There were however no cases found in which sirolimus was used post–CAR T-cell therapy. Everolimus was reintroduced in 1 case, 450 days post–CAR T-cell therapy, with that case reportedly in remission at 25 months follow-up.^27^ Although the paucity of larger scale data pertaining to the effects of mTOR inhibition on CAR T-cell therapy efficacy prevent definitive recommendations, at the time of writing if maintenance immunosuppression is to be reintroduced or maintained throughout CAR T treatment, mTOR inhibitors may be the more favorable of existing options as compared with CNIs, antimetabolites, or corticosteroids.

#### Belatacept

Belatacept is a fusion protein designed to block the CD28-activating receptor on T cells by binding to its B7-1 and B7-2 ligands.^63^ This mechanism of action makes suppression of CAR T cells likely, and with the additional concern for increased PTLD incidence in EBV-seronegative recipients of EBV-seropositive donor organs, makes belatacept an unlikely suitable choice for maintenance immunosuppression in solid organ transplant recipients following CAR T-cell treatment. In our systematic review of 44 reported cases of CAR T-cell therapy for PTLD in solid organ transplant recipients, belatacept was not used in any case; to our knowledge, there are no published clinical reports describing belatacept as maintenance immunosuppression following CAR T-cell therapy for PTLD.

#### Hypogammaglobulinemia

CD19-directed CAR T-cell therapy commonly results in on-target B cell aplasia with secondary hypogammaglobulinemia, which may persist for months to years and can contribute to late bacterial infections.^64^^,^^65^ Although the principle is similar to other therapy-associated secondary antibody deficiencies (e.g., anti-CD38–based therapy in myeloma), CD19 CAR T-cell–associated humoral immunodeficiency can be profound and prolonged. It is therefore suggested that systematic IgG monitoring and a low threshold for replacement are appropriate in high-risk patients, which we suggest would include those with PTLD after solid organ transplantation.^64^ The ASTCT recommend measuring serum IgG before lymphodepletion and then at least monthly for the first 3 months, with ongoing surveillance thereafter in patients with persistent B-cell aplasia or recurrent infections. In adults, routine prophylactic i.v. Ig for asymptomatic patients remains variable across centers. Expert guidance supports i.v. Ig for IgG ≤ 400 mg/dl, particularly in the first 3 months after infusion and/or in the presence of recurrent or severe bacterial infections. Replacement for IgG 400–600 mg/dl can also be considered when infections are recurrent.^64^

#### Engineering CAR T Cells to be Resistant to Immunosuppression

Alterations to CAR T-cell sensitivity to ciclosporin A by introducing a mutation in calcineurin subunit A allowed improved effector function *in vitro* and antitumor effects in a xenograft mouse model in the presence of ciclosporin A compared with CAR T cells without these modifications.^66^ Maldini *et al.*^67^ demonstrated that genetic knockout of FK506-binding protein 1A in humanized mice CAR T cells conferred resistance to rapamycin and tacrolimus and allowed persistence and tumor progression control. In addition, exogenous immunosuppression appears to facilitate the management of allorejection of “off the shelf” CAR T cells.^68^

Although allogenic sources of CAR T are challenged by graft versus host disease and host versus graft rejection, engineered approaches such as these may be particularly pertinent to maintenance immunosuppression in solid organ transplants. This could allow a more bespoke, personalized approach by selectively removing the suppressive effect of exogenous immunosuppressive agents on the CAR T cell through mechanistic pathway manipulation. However, one must be cognizant that more potent and persistent CAR T-cell function through genetic modification synchronous with organ selective exogenous immunosuppression may predispose to increased infection through overall intensified immunosuppressive burden.

#### Cancer Treatment Response Versus Immunosuppression

In the presence of persistent disease, an assessment of current CAR T-cell activity is important and though standard T-cell panels can be used as surrogate markers of CAR T persistence, specific quantifying methods are increasingly available.^69^ In the presence of persistent CAR T-cell activity in the face of persistent or relapsed disease, exogenous immunosuppression of any pathway is likely to impair anticancer activity of the CAR T-cells to some extent, and increase the likelihood of disease-related mortality. In addition, persistent CAR T-cell activity will likely result in persistent humoral immunosuppression because of B cell depletion, despite cancer persistence; a fact that could result in severe infection and sepsis with the reintroduction of solid organ transplant–directed immunosuppression. In persistent disease, continued withholding of immunosuppression with close monitoring may be necessary; however, maintaining allograft function through the minimization of allograft rejection risk may facilitate a broader armamentarium of chemotherapeutic options.

### In our Practice

**Figure 2:**
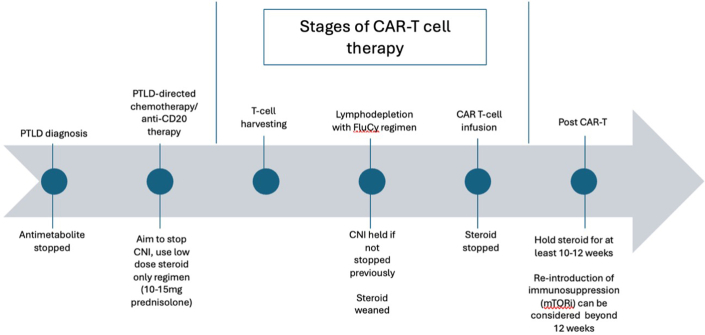
Outline of current immunosuppression management in our institution before, during, and after CAR T-cell therapy for post-transplant lymphoproliferative disorders. CAR, chimeric antigen receptor; CAR-T, CAR T-cell therapy; CNI calcineurin inhibitors; mTORi, mammalian target of rapamycin inhibitors; PTLD, posttransplant lymphoproliferative disorder.

Although these nuanced decisions are challenging because of a lack of robust prospective data, and lack of consensus on optimal management, the following represents some practice considerations informed by our own limited institutional experience with CAR T-cell therapy for PTLD in kidney transplant recipients (Figure 2).

At the time of PTLD diagnosis, we aim to cease the antimetabolite. Following initiation of PTLD-directed chemotherapy and anti-CD20 therapy, we aim, where clinically feasible, to withhold CNI therapy and continue a single-agent low-dose corticosteroid maintenance regimen (typically 10–15 mg prednisolone daily) as a bridge strategy, with the intention to taper and discontinue corticosteroids completely before CAR T-cell infusion, where possible.

For patients proceeding to CAR T-cell therapy, we aim to withhold CNI before T cell harvesting. If a corticosteroid-only maintenance regimen is not achievable before leukapheresis, CNI is instead discontinued at the start of lymphodepleting chemotherapy. This approach reflects the known ability of CNIs to suppress T-cell activation and proliferation, which could theoretically attenuate CAR T-cell expansion and function if continued through leukapheresis or lymphodepletion.

In relation to corticosteroids, our goal is a steroid-minimizing (ideally steroid-free) protocol at the time of CAR T-cell infusion and for approximately 10 to 12 weeks postinfusion, with close monitoring, to allow CAR T cells to activate, proliferate, and expand without the potential suppressive effects of exogenous corticosteroid exposure. Where chronic corticosteroid therapy has been used, the risk of adrenal suppression and steroid dependency in solid organ transplant recipients must be appreciated during dose reduction or withdrawal. Adrenal function can be assessed in the period before CAR T-cell infusion with morning cortisol measurements and, where indicated, synacthen testing, followed by judicious weaning to 0.^71^ Importantly, if toxicities such as cytokine release syndrome or immune effector cell–associated neurotoxicity syndrome occur, these are managed according to standard protocols, including corticosteroids or IL-6 antagonists where indicated, independent of solid organ transplant maintenance corticosteroid considerations, and are weaned appropriately thereafter along standard protocols.

During the period of exogenous immunosuppression reduction or withdrawal, routine monitoring involves serial blood tests and trending of donor-specific antibodies from baseline titers, T- and B-cell subset counts, allograft function, and proteinuria. Where CAR T-cell treatment response is favorable and clinical remission is induced, longer-term maintenance immunosuppression beyond 10 to 12 weeks should be individualized, taking into account the persistence of CAR T cells and CAR T-cell therapy–mediated ongoing immunosuppression, the risk of relapse potentially related to exogenous immunosuppression, the evolving risk of sepsis, and the competing risk of allograft rejection as time from CAR T-cell infusion elapses. In cases where remission is induced and reinstitution of maintenance immunosuppression is considered, mechanistic and translational literature suggests that mTOR inhibition may be a theoretically favorably initial approach among possible immunosuppression options. However, clinical data in this specific clinical setting remain limited, and some individual patients may experience prolonged periods of stable allograft function in the absence of solid organ immunosuppression following CAR T-cell treatment.

CAR T-cell therapy is increasingly indicated earlier in the PTLD treatment paradigm, rather than following multiple cycles of intensive chemotherapy, which represents an important consideration for future prospective studies and influence infection and allograft rejection risks. Despite its rarity, PTLD, with its subtype heterogeneity and diverse etiological risk factors, requires further study to establish optimal therapeutic regimens.

## Conclusion

Immunosuppression management in solid organ transplant recipients before and after CAR T-cell therapy is complex and must be individualized; however, the limited available evidence in this setting restricts truly personalized decision-making. Clinicians must balance the risks of CAR T-cell treatment failure or disease relapse, secondary malignancy, and life-threatening infection against each patient’s immunological risk of allograft rejection.

Future directions include incorporating more granular measures of immune reconstitution and CAR T-cell biology. These include assessments of CAR T-cell therapy efficacy, persistence quantification, extent of ongoing B-cell aplasia, and degree of treatment-induced lymphopenia. In parallel, emerging noninvasive biomarkers of subclinical allograft injury and rejection risk, including donor-derived cell-free DNA, may help refine immunosuppression treatment algorithms and improve outcomes.^70^

## Disclosure

All the authors declared no competing interests.

## Supplementary Material

Supplementary File (Word) PRISMA Checklist 2020.
